# Comparing Perioperative Outcomes of Choledochoduodenostomy and Roux-en-Y Hepaticojejunostomy in Difficult Choledocholithiasis: The Experience of a Brazilian Tertiary-Level University Hospital

**DOI:** 10.7759/cureus.98025

**Published:** 2025-11-28

**Authors:** Otávio B Ceribeli, Philipe F Tafner, Juan Mariano H Aguilar, João Victor Franqueira, Aléxis Vinicius Q Santos, Ana Luisa T Vaz, Martinho A Gestic, Murillo P Utrini, Francisco Callejas-Neto, Everton Cazzo

**Affiliations:** 1 Surgery, Faculty of Medical Sciences, Universidade Estadual de Campinas (UNICAMP), Campinas, BRA; 2 Surgery, Universidade Nove de Julho (UNINOVE), São Paulo, BRA

**Keywords:** biliary tract surgical procedures, choledochoduodenostomy, choledocholithiasis, hepaticojejunostomy, morbidity

## Abstract

Introduction: Choledocholithiasis may cause cholangitis, pancreatitis, or liver failure. Although endoscopic retrograde cholangiopancreatography (ERCP) is the standard treatment, it may fail or be unavailable, making biliary-enteric anastomoses such as choledochoduodenostomy (CDD) and Roux-en-Y hepaticojejunostomy (HJ-RY) important surgical alternatives.

Methods: This single-center retrospective study (2011-2023) included 46 patients who underwent CDD or HJ-RY for choledocholithiasis, either as primary treatment or after failed/unavailable ERCP. The primary endpoint was 30-day morbidity; secondary endpoints included severe morbidity (Clavien-Dindo ≥3) and mortality. Clinical, laboratory, operative, and postoperative variables were analyzed. Multivariable logistic regression identified independent predictors of 30-day morbidity, severe morbidity, and mortality.

Results: The mean age was 53 years, with 58.7% females. Prior cholangitis occurred in 47.2% and previous ERCP in 60.9% of cases. Compared with HJ-RY, CDD was performed in older patients (58 ± 15 vs. 45 ± 17 years; p=0.039) and those with larger bile ducts (2.1 ± 0.4 vs. 1.6 ± 0.6 cm; p=0.005). No significant differences were found between techniques or between patients with and without prior ERCP in morbidity, severe complications, or mortality. In multivariable analysis, no variable independently predicted 30-day morbidity or mortality, though cholangitis showed a trend toward higher risk and bile duct diameter toward protection.

Conclusion: CDD and HJ-RY are safe and effective options for choledocholithiasis, with comparable short-term outcomes. The choice should rely on anatomical and intraoperative factors rather than expected differences in perioperative morbidity.

## Introduction

Choledocholithiasis is a common condition that can lead to cholangitis, biliary pancreatitis, and liver failure, requiring definitive treatment. Currently, endoscopic retrograde cholangiopancreatography (ERCP) with sphincterotomy is considered the gold standard for most patients [1]. This approach achieves high rates of duct clearance, carries a low risk of severe complications, and is associated with lower morbidity compared with surgery.

However, ERCP may fail or be contraindicated in certain situations, such as in patients with large, multiple, or impacted stones, altered anatomy, or previous upper gastrointestinal surgery (e.g., Roux-en-Y gastric bypass), or may be unavailable in regional hospitals with limited resources. In these scenarios, surgical biliary-enteric anastomosis remains a definitive therapeutic option for biliary decompression.

Among the available techniques, choledochoduodenostomy (CDD) and Roux-en-Y hepaticojejunostomy (HJ-RY) are the most widely employed. The choice between them depends on anatomical, technical, and patient-related factors. In general, HJ-RY is preferred when duodenal mobilization is difficult or in patients with prior gastric or duodenal surgery that precludes safe access to the duodenum, whereas CDD is often considered a simpler alternative and maintains the advantage of facilitating future endoscopic access to the biliary tract [2-4].

At our institution, the selection of the surgical approach follows a non-mandatory protocol designed to standardize decision-making while allowing for clinical judgment. CDD is typically favored in patients with a dilated common bile duct (≥2.0 cm) and a mobile, accessible duodenum without previous foregut reconstruction. Conversely, HJ-RY is generally indicated when the bile duct is smaller, duodenal mobilization is technically unfavorable, or prior surgeries (e.g., Billroth II, Roux-en-Y gastric bypass) preclude safe duodenal anastomosis. Nevertheless, final operative decisions may vary according to the surgeon’s preference, intraoperative findings, and specific patient characteristics, reflecting the pragmatic nature of real-world surgical practice.

The long-term complications differ between these two techniques. CDD may be associated with sump syndrome, biliary reflux, and recurrent cholangitis [5-7], whereas HJ-RY, despite mitigating reflux, may present a higher risk of anastomotic stricture and challenges for endoscopic management [3,4]. Although some studies have suggested a potential association between chronic biliary reflux and an increased risk of biliary tract malignancy, these observations are based on limited and heterogeneous evidence and should be interpreted as hypothesis-generating rather than conclusive findings [8]. Our study did not evaluate malignancy outcomes, and any reference to this topic is included solely for contextual discussion.

Given these considerations, evaluating short-term outcomes while accounting for relevant anatomical and clinical factors is essential to guide surgical decision-making. The aim of this study was to compare clinical, laboratory, and surgical outcomes between choledochoduodenostomy and Roux-en-Y hepaticojejunostomy performed for choledocholithiasis, either as primary treatment or after ERCP failure or unavailability, using multivariable analysis to adjust for potential confounders.

## Materials and methods

This was a retrospective, single-center study based on the review of medical records of patients who underwent biliary-enteric anastomosis for choledocholithiasis between 2011 and 2023 at a tertiary-level university referral hospital, Hospital de Clínicas da Universidade Estadual de Campinas (UNICAMP), Campinas, São Paulo, Brazil. The study was approved by the Ethics Committee, UNICAMP (approval number: 6.552.262).

Study population

All adult patients (≥18 years) who consecutively underwent biliary-enteric anastomosis for choledocholithiasis between January 2011 and December 2023 were included, identified through the institution’s electronic medical record system. Both patients operated on as primary (upfront) treatment and those undergoing surgery after failure or unavailability of ERCP were eligible. Patients who underwent biliary-enteric anastomosis for other indications, such as malignant neoplasms of the biliary tract or pancreas, iatrogenic biliary injury after cholecystectomy, or anastomotic stricture following liver transplantation, were excluded. Cases with incomplete data precluding adequate clinical or surgical assessment were also excluded.

During the study period, 54 patients underwent biliary-enteric anastomosis for choledocholithiasis. After excluding eight with incomplete data, the final sample comprised 46 consecutive patients with confirmed diagnoses, treated surgically either as a primary indication or after failed or unavailable ERCP. Patients were classified according to the surgical technique performed: CDD or HJ-RY.

Variables and study outcomes

Demographic and clinical data were collected, including age, sex, and presence of relevant comorbidities, as well as intraoperative variables such as total operative time and estimated blood loss. In the immediate postoperative period, the length of stay in the intensive care unit (ICU), duration of hospital stay, and occurrence of complications such as enteric fistula, cholangitis, hepatic abscess, abdominal collections, or acute renal failure were analyzed. Postoperative complications were identified through systematic chart review covering the first 30 postoperative days, including outpatient follow-up records and readmission documentation. Complications were stratified according to severity through the Clavien-Dindo grading system [9]. Laboratory tests (bilirubin levels, transaminases, alkaline phosphatase, and white blood cell and platelet count) were also evaluated, as well as stone characteristics, including number and mean size, and duct size according to the operative report.

The primary endpoint was 30-day overall morbidity (any postoperative complication within 30 days of surgery, Clavien-Dindo I-V). Secondary endpoints included severe morbidity (Clavien-Dindo ≥III) and 30-day mortality.

Surgical technique

Procedures were performed by laparotomy (Chevron or Kocher incision) or laparoscopy, according to the surgeon's preference and anatomical feasibility. The usual indication for CDD included a dilated common bile duct (≥2.0 cm) and a mobile, accessible duodenum without prior gastric or duodenal surgery. HJ-RY was typically indicated when the bile duct was narrow (<2.0 cm), duodenal mobilization was technically unfavorable, or prior reconstruction (e.g., Billroth II or Roux-en-Y gastric bypass) precluded safe duodenal access. These institutional criteria served as general guidance rather than mandatory rules; in some cases, the final decision depended on the surgeon’s intraoperative judgment and patient-specific anatomical conditions.

Statistical analysis

Categorical variables were compared using Pearson’s chi-square or Fisher’s exact test, as appropriate. Continuous variables were assessed using the Mann-Whitney U test or Student’s t-test, depending on data distribution. Missing data were handled by complete-case analysis, as the proportion of missing values was small and imputation was not performed due to sample size constraints. Normality was verified with the Shapiro-Wilk test. Continuous variables were expressed as mean ± standard deviation or median (interquartile range), as appropriate. Variables associated with 30-day morbidity or mortality in univariable analyses (p ≤ 0.25) were included in a multivariable logistic regression model to identify independent predictors of each outcome. Results were reported as odds ratios (OR) with 95% confidence intervals (95% CI). Statistical significance was set at p < 0.05. All analyses were performed using IBM SPSS Statistics for Windows, version 29.0 (Released 2021; IBM Corp., Armonk, New York, United States).

## Results

The sample showed a predominance of female patients, with an overall median age of 54.5 years (IQR, 42.2-64.5). Most cases were admitted electively through the outpatient clinic, although a significant proportion were referred from emergency care. There was a high frequency of previous cholangitis, present in more than half of the patients, as well as prior ERCP, observed in approximately 40% of cases. The median follow-up time was 14.5 months (range, 1-24 months). The general clinical characteristics of the study population are summarized in Table 1.

**Table 1:** General characteristics of the study population ALT: alanine aminotransferase; ALP: alkaline phosphatase; AST: aspartate aminotransferase; BMI: body mass index; ERCP: endoscopic retrograde cholangiopancreatography; GGT: gamma-glutamyl transferase; ICU: intensive care unit; INR: international normalized ratio; IQR: interquartile range; WBC: white blood cell count

Characteristics		Values
Sex, n (%)		
Female		27 (58.7%)
Male		19 (41.3%)
Comorbidities, n (%)		
Hypertension, n (%)		16 (34.8%)
Diabetes, n (%)		11 (23.9%)
Heart Disease, n (%)		1 (2.2%)
Smoking, n (%)		
Current		9 (19.6%)
Former		4 (8.7%)
Alcohol use, n (%)		
Current		2 (4.4%)
Former		5 (10.9%)
Admission source, n (%)		
Emergency		17 (37%)
Outpatient		29 (63%)
Symptoms, n (%)		
Abdominal pain		46 (100%)
Weight Loss		11 (23.9%)
Fever		20 (43.5%)
Jaundice		41 (89.1%)
Past history, n (%)		
Pancreatitis		6 (13%)
Cholangitis		22 (47.2%)
ECRP		28 (60.9%)
Cholecystectomy		26 (56.5%)
ERCP complications, n (%)		
Stent migration		2 (4.4%)
Pancreatitis		2 (4.4%)
Papillary laceration		1 (2.2%)
Duodenal perforation		1 (2.2%)
Admission labs, mean±SD		
WBC (x10⁹/L)		8.0 ± 3.0
Hemoglobin (g/dL)		13.1 ± 1.5
Hematocrit (%)		40.2 ± 4.7
AST (U/L)		89.7 ± 81.9
ALT (U/L)		89.9 ± 83.6
ALP (U/L)		450.1 ± 304.9
GGT (U/L)		655.3 ± 680.8
Total bilirubin (mg/dL)		3.0 ± 4.0
Preoperative labs, mean±SD		
WBC (x10⁹/L)		7.9 ± 4.0
Hemoglobin (g/dL)		13.0 ± 1.9
Hematocrit (%)		37.9 ± 5.4
AST (U/L)		497.6 ± 2785.6
ALT (U/L)		185.3 ± 826.0
ALP (U/L)		229.4 ± 239.3
GGT (U/L)		269.7 ± 352.0
Total bilirubin (mg/dL)		1.2 ± 1.6
INR		1.10 ± 0.18
Creatinine (mg/dL)		0.79 ± 0.22
Platelets (x10⁹/L)		248.0 ± 78.6
Stone Characteristics		
Number of Stones, n (%)	Single	10 (21.7%)
	Multiple	36 (78.3%)
Largest stone size (cm), mean±SD		1.5 ± 1.1
Bile duct diameter (cm), mean±SD		1.8 ± 0.9
Operative data		
Surgical Approach, n (%)	Open	44 (95.7%)
	Laparoscopic	2 (4.3%)
Estimated blood loss (mL), mean±SD		440 ± 289
Operative time (min), mean±SD		226 ± 56
Hospital stay (days), mean±SD		11.3 ± 10.8
ICU stay (days), mean±SD		1.1 ± 1.9
Surgical complications, n (%)		
Overall		12 (26.1%)
Bile leak		4 (8.7%)
Intestinal Obstruction		1 (2.2%)
Prolonged ileus		2 (4.3%)
Pneumonia		2 (4.3%)
Clavien–Dindo <3		7 (15.2%)
Clavien–Dindo ≥3		5 (10.9%)
Mortality – n (%)		4 (8.7%)

Of the 46 patients included in the study, who underwent surgical management, the most common reasons were ERCP failure (n=28; 60.9%), previous surgery with duodenal exclusion (n=6; 13.0%), or intrahepatic stones (n=4; 8.7%). Other indications for upfront surgery without an ERCP attempt included large stones (n=3; 6.5%), post-cholecystectomy strictures (n=2; 4.3%), and ERCP unavailability (n=3; 6.5%).

In the comparison between groups (CDD vs. HJ-RY), shown in Table 2, age was significantly higher in the CDD group (58 ± 15 years) compared with the HJ-RY group (45 ± 17 years; p=0.039). There was no significant difference in sex distribution (p=0.359). Among comorbidities, hypertension and diabetes were more frequent in the CDD group, but without statistical significance. Regarding symptoms, abdominal pain was present in all patients. Weight loss (n=7; 24.1% vs. n=4; 23.5%; p=1.000) and jaundice (n=26; 89.7% vs. n=15; 88.2%; p=1.000) occurred in similar proportions. Fever was more common in the HJ-RY group (n=9; 31.0% vs. n=11; 64.7%; p=0.025. With respect to past history, cholangitis was also more prevalent in the HJ-RY group (n=11; 37.9% vs. n=11; 64.7%), with a trend toward significance (p=0.063), whereas previous pancreatitis was comparably less common in both groups (n=3; 10.3% vs. n=3; 17.6%; p=0.48). Prior ERCP was more common in the CDD group (n=19; 51.7% vs. n=11; 64.7%; p=0.36), without statistical significance. Preoperative laboratory analysis showed no significant differences between groups in white blood cell count, hemoglobin, hematocrit, liver enzymes, total bilirubin, INR, creatinine, or platelet count.

**Table 2:** Comparison between patients undergoing choledochoduodenostomy vs. Roux-en-Y hepaticojejunostomy Statistical tests: * = Student's t (mean ± standard deviation; normal distribution); † = Mann–Whitney U test (median [interquartile range]; non-normal distribution); ‡ = Pearson's chi-square; § = Fisher's exact. CDD: Choledochoduodenostomy; HJ-YR: Roux-en-Y hepaticojejunostomy; ALT: alanine aminotransferase; ALP: alkaline phosphatase; AST: aspartate aminotransferase; BMI: body mass index; ERCP: endoscopic retrograde cholangiopancreatography; GGT: gamma-glutamyl transferase; ICU: intensive care unit; INR: international normalized ratio; IQR: interquartile range; WBC: white blood cell count; NA: not applicable.

Variable	CDD (n=29)	HJ-RY (n=17)	Statistic	P-value
Demographic and Clinical Data				
Age (years), mean±SD	58 ± 15	45 ± 17	U = 155.5	0.039†
Sex (male), n (%)	10 (34.5%)	9 (52.9%)	χ² = 1.51	0.219‡
Hypertension, n (%)	12 (41.4%)	4 (23.5%)	χ² ≈ 1.39	0.338‡
Diabetes, n (%)	9 (31.0%)	2 (11.8%)	χ² = 2.20	0.138‡
Heart disease, n (%)	1 (3.4%)	0 (0.0%)	NA	1.000§
Origin, n (%)				
Outpatient	19 (65.5%)	10 (58.8%)	χ² = 0.21	0.891‡
Emergency unit	10 (35.5%)	7 (42.2%)		
Symptoms, n (%)				
Abdominal pain	29 (100.0%)	17 (100.0%)	NA	1.000§
Weight loss	7 (24.1%)	4 (23.5%)	χ² ≈ 0.00	0.96‡
Jaundice	26 (89.7%)	15 (88.2%)	χ² ≈ 0.02	0.89‡
Fever	9 (31.0%)	11 (64.7%)	χ² ≈ 5.02	0.025‡
Medical History, n (%)				
Cholangitis	11 (37.9%)	11 (64.7%)	χ² ≈ 3.45	0.063‡
Pancreatitis	3 (10.3%)	3 (17.6%)	χ² ≈ 0.51	0.48‡
Previous ERCP	19 (65.5%)	9 (52.9%)	χ² ≈ 0.73	0.39‡
Previous cholecystectomy	15 (51.7%)	11 (64.7%)	χ² ≈ 0.83	0.36‡
Characteristics of Stones and Bile Duct				
Largest stone size (cm), median (IQR)	1.2 (0.8–1.8)	1.0 (0.9–1.5)	U ≈ 230	0.767†
Number of stones, n (%)	Single: 7 (24.1%)/Multiple: 12 (75.9%)	Single: 3 (17.6%)/Multiple: 14 (82.4%)	χ² = 0.27	0.723‡
Bile duct diameter (cm), mean±SD	2.1 ± 0.4	1.6 ± 0.6	t ≈ 3.11	0.005*
Preoperative Tests, mean±SD				
WBC (10^3^ cells/mm³)	7.3 ± 2.6	9.2 ± 3.5	t ≈ 1.46	0.155*
Hemoglobin (g/dL)	13.29 ± 1.67	12.86 ± 1.27	t ≈ 0.75	0.459*
Hematocrit (%)	41.16 ± 5.52	38.75 ± 3.02	t ≈ 1.29	0.204*
AST (U/L)	72.26 ± 73.69	115.92 ± 89.60	U ≈ 190	0.162†
ALT (U/L)	81.98 ± 79.48	102.33 ± 91.86	U ≈ 200	0.612†
ALP (U/L)	383.59 ± 273.84	552.82 ± 334.60	U ≈ 180	0.178†
GGT (U/L)	674.50 ± 779.27	620.45 ± 484.39	U ≈ 215	0.635†
Total bilirubin (mg/dL)	3.03 ± 4.51	2.92 ± 3.13	U ≈ 210	0.586†
INR	1.10 ± 0.21	1.09 ± 0.14	t ≈ 0.04	0.972*
Creatinine (mg/dL)	0.79 ± 0.19	0.78 ± 0.27	t ≈ 0.10	0.921*
Platelets (10^6^/mm³)	244.8 ± 74.1	253.5 ± 87.9	t ≈ 0.34	0.736*
Operative Data				
Operative time (min), mean±SD	205.7 ± 58.6	250.0 ± 45.2	t ≈ 1.47	0.153*
Estimated blood loss (mL), mean±SD	390.9 ± 259.6	500.0 ± 326.9	U ≈ 210	0.429†
Length of hospital stay (days), median (IQR)	8.5 (6.8–11.0)	9.0 (6.5–12.0)	U ≈ 230	0.769†
ICU stay (days), median (IQR)	0.0 (0.0–1.2)	0.0 (0.0–2.0)	U ≈ 220	0.565†
Surgical approach (laparoscopy), n (%)	2 (6.9%)	0 (0.0%)	NA	0.524§
Morbidity and Mortality, n (%)				
Complications (any), n (%)	7 (24.1%)	5 (29.4%)	χ² ≈ 0.04	0.964‡
Clavien-Dindo ≥ 3 complications, n (%)	3 (10.3%)	2 (11.8%)	χ² = 0.02	1.000‡
Mortality, n (%)	2 (6.9%)	2 (11.8%)	χ² = 0.32	0.619‡

Stone and bile duct characteristics are described in Table 2. The bile duct diameter was significantly larger in patients undergoing CDD (2.1 ± 0.4 cm) compared with those treated with HJ-RY (1.6 ± 0.6 cm; p=0.005). The number and size of stones did not differ between groups. Regarding operative parameters, there was a tendency toward longer operative time in the HJ-RY group (250.0 ± 45.2 minutes vs. 205.7 ± 58.6 minutes; p=0.153) and no significant difference in estimated blood loss (500.0 ± 326.9 mL vs. 390.9 ± 259.6 mL; p=0.429). Hospital and ICU stays were similar between groups. As for morbidity and mortality, the complication rate was similar (24.1% in the CDD group vs. 29.4% in the HJ-RY group; p=0.964). Severe complications (Clavien-Dindo ≥3) occurred in comparable proportions (10.3% vs. 11.8%; p=1.000), as did mortality (6.9% vs. 11.8%; p=0.619).

In the multivariable logistic regression analysis evaluating factors associated with 30-day morbidity (Table 3), none of the assessed variables reached statistical significance. Although not significant, the presence of cholangitis was associated with higher odds of postoperative complications (OR = 3.08; 95%CI 0.52-18.25; p = 0.212). A trend toward lower morbidity was observed with increasing bile duct diameter (OR = 0.31; 95% CI 0.05-3.46; p = 0.179), suggesting a potential protective effect in patients with more dilated ducts. Neither age, diabetes, fever, previous ERCP, operative time, nor admission source was independently related to 30-day morbidity. The observed results were similar, considering severe 30-day morbidity (Table 4). Similarly, for 30-day mortality (Table 5), no variable showed a statistically significant association. The presence of cholangitis demonstrated the highest odds ratio (OR = 6.09; 95%CI 0.40-93.51; p = 0.189), suggesting a possible trend toward increased risk, though this did not reach significance. A similar inverse, non-significant trend was observed for bile duct diameter (OR = 0.23; 95%CI 0.02-2.89; p = 0.165). Other factors, including age, diabetes, fever, previous ERCP, operative time, and admission source, were not independently associated with postoperative mortality.

**Table 3:** Multivariable analysis — 30-day morbidity All results provided by logistic regression (Wald) ERCP: endoscopic retrograde cholangiopancreatography

Variable	Coefficient of Regression	OR (95% CI)	P-Value
Age	0.012	1.01 (0.96–1.07)	0.745
Diabetes	0.582	1.79 (0.24–13.11)	0.564
Fever	0.403	1.50 (0.25–9.06)	0.662
Cholangitis	1.124	3.08 (0.52–18.25)	0.212
Previous ERCP	0.198	1.22 (0.20–7.27)	0.828
Operative time	-0.056	0.95 (0.63–1.44)	0.824
Bile duct diameter	-1.118	0.31 (0.05–3.46)	0.179
Admission: Emergency vs. Outpatient	0.386	1.47 (0.18–12.20)	0.721

**Table 4:** Multivariable analysis — severe morbidity (CD≥3) All results provided by logistic regression (Wald). ERCP: endoscopic retrograde cholangiopancreatography; CD: Clavien-Dindo

Variable	Coefficient of Regression	OR (95% CI)	P-value
Age	0.029	1.03 (0.95–1.12)	0.478
Diabetes	-0.217	0.80 (0.07–8.79)	0.857
Fever	0.946	2.58 (0.21–31.39)	0.448
Cholangitis	1.526	4.60 (0.34–61.38)	0.253
Previous ERCP	0.583	1.79 (0.17–18.63)	0.625
Bile duct diameter	-1.120	0.51 (0.22-5.7)	0.249
Operative time	0.120	1.13 (0.44–2.92)	0.801
Admission: Emergency vs Outpatient	0.739	2.09 (0.17–25.03)	0.568

**Table 5:** Multivariable analysis — 30-day mortality All results provided by logistic regression (Wald). ERCP: endoscopic retrograde cholangiopancreatography; CD: Clavien-Dindo

Variable	Coefficient of Regression	OR (95% CI)	P-value
Age	0.034	1.03 (0.95–1.12)	0.463
Diabetes	-0.693	0.50 (0.03–8.70)	0.639
Fever	1.063	2.90 (0.22–38.00)	0.417
Cholangitis	1.807	6.09 (0.40–93.51)	0.189
Previous ERCP	0.707	2.03 (0.18–23.31)	0.568
Operative time	0.089	1.09 (0.49–2.43)	0.835
Bile duct diameter	-1.458	0.23 (0.02–2.89)	0.165
Admission: Emergency vs Outpatient	0.572	1.77 (0.12–26.86)	0.677

Regarding the comparison between patients with prior ERCP and those who underwent upfront surgery, there were no significant differences in demographic characteristics, clinical presentation, operative variables, or postoperative outcomes, including morbidity, severe complications, and mortality rates (Table 6).

**Table 6:** Comparison between patients with previous ERCP and those undergoing upfront surgery * = Student’s t-test † = Mann–Whitney U test ‡ = χ² test § = Fisher’s exact test. ERCP: endoscopic retrograde cholangiopancreatography; ICU: intensive care unit; HJ-RY: Roux-en-Y hepaticojejunostomy; CDD: choledochoduodenostomy

Variable	Prior ERCP (N = 28)	Upfront surgery (N = 18)	Statistic	P-value
Demographic and Clinical Data				
Age (years), median (IQR)	55 (46.5-65.2)	51.5 (42-62.8)	t ≈ 0.62	0.540*
Sex (male), n (%)	12 (42.9%)	8 (44.4%)	χ² ≈ 0.01	0.937‡
Hypertension, n (%)	10 (35.7%)	5 (27.8%)	χ² ≈ 0.28	0.598‡
Diabetes, n (%)	6 (21.4%)	5 (27.8%)	χ² ≈ 0.21	0.650‡
Heart disease, n (%)	1 (3.6%)	0 (0.0%)	NA	1.000§
Origin, n (%)				
Outpatient	17 (60.7%)	12 (66.7%)	χ² ≈ 0.17	0.683‡
Emergency unit	11 (39.3%)	6 (33.3%)		
Symptoms, n (%)				
Abdominal pain	28 (100.0%)	18 (100.0%)	NA	1.000§
Weight loss	8 (28.6%)	3 (16.7%)	NA	0.486§
Jaundice	25 (89.3%)	16 (88.9%)	NA	1.000§
Fever	13 (46.4%)	7 (38.9%)	χ² ≈ 0.25	0.615‡
Medical History, n (%)				
Cholangitis	13 (46.4%)	9 (50.0%)	χ² ≈ 0.06	0.813‡
Pancreatitis	5 (17.9%)	1 (5.6%)	NA	0.380§
Previous cholecystectomy	14 (50.0%)	9 (50.0%)	χ² ≈ 0.00	1.000‡
Characteristics of Stones and Bile Duct				
Largest stone size (cm), median (IQR)	1.1 (0.8–1.6)	1.2 (0.9–1.8)	U ≈ 175	0.628†
Number of stones, n (%)	Single: 7 (25%)	Single: 3 (16.7%)	χ² ≈ 0.43	0.514‡
	Multiple: 21 (75%)	Multiple: 15 (83.3%)		
Bile duct diameter (cm), mean±SD	1.9 ± 0.5	2.0 ± 0.6	t ≈ 0.59	0.559*
Operative Data				
Type of Surgery, n (%)	HJ-RY: 19 (65.5%)	HJ-RY: 10 (34.5%)	χ² ≈ 0.73	0.39‡
	CDD: 9 (52.9%)	CDD: 8 (47.1%)		
Operative time (minutes), mean±SD	220 ± 62	240 ± 55	t ≈ 1.04	0.304*
Estimated blood loss (mL), mean±SD	410 ± 280	480 ± 310	U ≈ 190	0.485†
Postoperative Course				
Length of hospital stay (days), median (IQR)	9.0 (6.5–11.0)	7.5 (7.0–8.8)	U ≈ 129.5	0.438†
ICU stay (days), median (IQR)	0.0 (0.0–1.5)	0.0 (0.0–2.0)	U ≈ 90.0	0.519†
Morbidity and Mortality				
Complications (any), n (%)	7 (28.0%)	5 (33.3%)	NA	0.736§
Clavien-Dindo ≥ 3 complications, n (%)	3 (10.7%)	1 (5.6%)	NA	1.000§
Mortality, n (%)	3 (10.7%)	1 (5.6%)	NA	1.000§

## Discussion

In our sample, we identified a predominance of female patients with a mean age around 53 years, a finding consistent with previous national and international studies describing a higher frequency of gallstone disease and its complications in middle-aged women [1,2]. Most cases originated from outpatient care, though a substantial proportion of emergency admissions was also observed, a pattern frequently reported in contemporary epidemiological analyses [3,4]. The high prevalence of previous cholangitis episodes (47.2%) and the considerable proportion of patients with a history of ERCP (60.9%) reflect the clinical complexity of this population and the central role of therapeutic endoscopy in the management of choledocholithiasis [1,3,7,10].

When comparing surgical techniques, significant differences were observed in age and bile duct diameter, both higher among patients undergoing CDD (58 ± 15 years and 2.1 ± 0.4 cm, respectively) compared with those submitted to HJ-RY (45 ± 17 years and 1.6 ± 0.6 cm; p = 0.039 and p = 0.005, respectively). These findings suggest that CDD tended to be employed in older patients with more dilated ducts, likely reflecting technical preference in anatomically favorable cases and the advantage of preserving endoscopic access for future interventions. The literature corroborates that anatomical and clinical characteristics are decisive in choosing the most appropriate biliary-enteric anastomosis, aiming to optimize outcomes and minimize complications [2,11-15].

In our multivariable analyses, none of the evaluated factors showed a statistically significant association with 30-day morbidity, severe morbidity (Clavien-Dindo ≥ III), or 30-day mortality. Nonetheless, certain trends were noteworthy. The presence of cholangitis consistently demonstrated higher odds of adverse outcomes in all models, with odds ratios of 3.08 for overall morbidity, 4.60 for severe morbidity, and 6.09 for 30-day mortality, although without statistical significance (p = 0.212, 0.253, and 0.189, respectively). These findings suggest that preoperative cholangitis may predispose patients to a more complex postoperative course, consistent with previous studies identifying biliary sepsis as a predictor of poor outcomes after biliary surgery [7,10,14].

Another relevant observation concerns the bile duct diameter, which showed an inverse relationship with morbidity and mortality, suggesting a possible protective effect. Larger duct calibers were associated with lower odds of 30-day morbidity (OR = 0.31; 95%CI 0.05-3.46) and mortality (OR = 0.23; 95%CI 0.02-2.89), indicating that a dilated bile duct may facilitate anastomotic construction and postoperative drainage, thereby reducing the likelihood of complications. Although these associations did not reach statistical significance, they align with prior findings that emphasize the technical advantages and lower anastomotic tension achieved in cases with more dilated ducts [2,12,16,17].

In this cohort, no independent associations between age, diabetes, fever, previous ERCP, operative time, or admission source and postoperative complications or mortality were demonstrated, although the study may have been underpowered to detect more modest effects. The absence of significant associations likely reflects the relatively small sample size, which limits the statistical power to detect subtle differences. Moreover, these results should be interpreted with caution, given the retrospective design, the heterogeneity of patient presentations, and the potential influence of unmeasured confounding factors.

In the context of long-term outcomes, CDD remains classically associated with sump syndrome, characterized by distal bile stasis leading to recurrent cholangitis, hepatic abscesses, and pancreatitis, as documented in long-term series [5-9,16-18]. HJ-RY, while avoiding duodenal reflux, may result in anastomotic strictures and recurrent cholangitis due to impaired bile flow or intestinal reflux [12-14,17-24]. Schreuder et al. [12] and others demonstrated similar overall morbidity between the two techniques but with distinct complication patterns.

Another practical aspect is endoscopic access. CDD allows easier future biliary interventions through ERCP, whereas HJ-RY often precludes conventional endoscopic access, requiring advanced techniques such as double-balloon enteroscopy or combined radiologic and laparoscopic approaches [25-28]. In our study, the selection of CDD for older patients with more dilated ducts likely reflected the anticipation of such future needs, consistent with the trend observed in recent clinical series [3,29,30].

Recent technological advances, including single-use cholangioscopy, endoscopic ultrasound-guided drainage, and laparo-endoscopic hybrid techniques, have expanded therapeutic options for managing late complications of biliary-enteric anastomoses. These tools reinforce that while the initial surgical choice remains relevant, future management increasingly relies on multidisciplinary strategies integrating surgery, advanced endoscopy, and minimally invasive technologies [28,29].

This study has several limitations that must be considered when interpreting the findings. The retrospective design and single-center nature inherently limit external generalizability. The decision between CDD and HJ-RY was inherently influenced by anatomical considerations and surgeon preference, which may have contributed to the similar outcomes observed between groups. The relatively small number of patients in each subgroup may have reduced the statistical power to identify subtle but clinically meaningful associations. Given the relatively small number of events (overall complications, severe complications, and deaths), the multivariable analyses performed were exploratory and underpowered, so that ORs, especially for cholangitis and bile duct diameter, should be interpreted with caution rather than as definitive predictors. Additionally, the institutional follow-up for benign biliary diseases is generally limited to one year, after which patients are discharged to primary or regional care due to institutional protocols. Consequently, long-term outcomes, particularly those related to late complications such as anastomotic stricture or sump syndrome, are difficult to determine. Variability in follow-up duration and the lack of standardized imaging or functional assessment further restrict the evaluation of late sequelae. Moreover, the absence of data on quality of life and endoscopic reinterventions limits a more comprehensive appraisal of the clinical impact of each surgical technique.

Despite these limitations, this study presents one of the more detailed institutional experiences with biliary-enteric anastomoses for benign disease, combining over a decade of data with consistent clinical, laboratory, and surgical documentation. Our findings reinforce that both CDD and HJ-RY offer comparable short-term safety when appropriately indicated. The observed trends suggest that preoperative cholangitis and smaller bile duct diameter may predispose to higher morbidity, warranting further investigation in larger, prospective cohorts.

Overall, the results highlight the need for individualized surgical planning that integrates anatomical and clinical factors, as well as the feasibility of future endoscopic reinterventions. The integration of modern endoscopic and hybrid surgical technologies may further refine the management of these complex patients, aiming to improve long-term outcomes while minimizing the risk of recurrent biliary complications.

## Conclusions

Both CDD and HJ-RY were safe and effective for the surgical management of choledocholithiasis, with no significant differences in perioperative morbidity or mortality. Patients undergoing CDD were older and had larger bile ducts, yet outcomes remained comparable. Prior ERCP did not influence operative complexity or postoperative results, underscoring that the choice of procedure should be guided by anatomical and intraoperative factors rather than concerns about short-term safety. These findings, however, primarily reflect short-term (30-day) outcomes; longer observation would be required to fully assess late complications such as sump syndrome or anastomotic strictures.
